# Costs, outcome and cost-effectiveness of neurocritical care: a multi-center observational study

**DOI:** 10.1186/s13054-018-2151-5

**Published:** 2018-09-20

**Authors:** R. Raj, S. Bendel, M. Reinikainen, S. Hoppu, R. Laitio, T. Ala-Kokko, S. Curtze, M. B. Skrifvars

**Affiliations:** 1 Department of Neurosurgery, Helsinki University Hospital and University of Helsinki, Topeliuksenkatu 5, PB 266, 00029 HUS Helsinki, Finland; 2 Department of Intensive Care, Kuopio University Hospital & University of Eastern Finland, Kuopio, Finland; 3 Department of Intensive Care, North Karelia Central Hospital, Joensuu, Finland; 4 Department of Intensive Care, Tampere University Hospital & University of Tampere, Tampere, Finland; 5 Department of Intensive Care, Turku University Hospital & University of Turku, Turku, Finland; 6 Department of Intensive Care, Oulu University Hospital & University of Oulu, Medical Research Center, Research Group of Surgery, Anesthesiology and Intensive Care, Oulu, Finland; 7 Department of Neurology, Helsinki University Hospital & University of Helsinki, Helsinki, Finland; 8 Department Anesthesia, Intensive Care and Pain Medicine and Department of Emergency Care and Services, Helsinki University Hospital & University of Helsinki, Helsinki, Finland

**Keywords:** Neurocritical care; neurointensive care; costs, Outcome; cost-effectiveness, Traumatic brain injury, Intracerebral hemorrhage, Acute ischemic stroke, Subarachnoid hemorrhage, Finland

## Abstract

**Background:**

Neurocritical illness is a growing healthcare problem with profound socioeconomic effects. We assessed differences in healthcare costs and long-term outcome for different forms of neurocritical illnesses treated in the intensive care unit (ICU).

**Methods:**

We used the prospective Finnish Intensive Care Consortium database to identify all adult patients treated for traumatic brain injury (TBI), intracerebral hemorrhage (ICH), subarachnoid hemorrhage (SAH) and acute ischemic stroke (AIS) at university hospital ICUs in Finland during 2003–2013. Outcome variables were one-year mortality and permanent disability. Total healthcare costs included the index university hospital costs, rehabilitation hospital costs and social security costs up to one year. All costs were converted to euros based on the 2013 currency rate.

**Results:**

In total 7044 patients were included (44% with TBI, 13% with ICH, 27% with SAH, 16% with AIS). In comparison to TBI, ICH was associated with the highest risk of death and permanent disability (OR 2.6, 95% CI 2.1–3.2 and OR 1.7, 95% CI 1.4–2.1), followed by AIS (OR 1.9, 95% CI 1.5–2.3 and OR 1.5, 95% CI 1.3–1.8) and SAH (OR 1.8, 95% CI 1.5–2.1 and OR 0.8, 95% CI 0.6–0.9), after adjusting for severity of illness. SAH was associated with the highest mean total costs (€51,906) followed by ICH (€47,661), TBI (€43,916) and AIS (€39,222). Cost per independent survivor was lower for TBI (€58,497) and SAH (€96,369) compared to AIS (€104,374) and ICH (€178,071).

**Conclusion:**

Neurocritical illnesses are costly and resource-demanding diseases associated with poor outcomes. Intensive care of patients with TBI or SAH more commonly result in independent survivors and is associated with lower total treatments costs compared to ICH and AIS.

**Electronic supplementary material:**

The online version of this article (10.1186/s13054-018-2151-5) contains supplementary material, which is available to authorized users.

## Background

Neurocritical illnesses, including traumatic brain injury (TBI), intracerebral hemorrhage (ICH), subarachnoid hemorrhage (SAH) and acute ischemic stroke (AIS), are major killers and impose a growing socioeconomic burden around the world [1–4]. The most severe illnesses or injuries are treated at specialized intensive care units (ICU). Intensive care is, however, very resource-demanding, comprising for example almost 0.7% of the national gross domestic product in the USA [5]. Furthermore, the need costs of intensive care beds appears to continue to rise [5–8]. Due to limited healthcare budgets clinicians and hospital administrators are interested in measuring and evaluating intensive care cost-effectiveness. Patients with neurocritical illnesses constitute approximately 20–25% of severely ill patients requiring intensive care (unpublished data from the Finnish Intensive Care Consortium). Still, cost-analysis studies of neurocritical illnesses are lacking [9].

Finland offers a unique setting to comprehensively assess healthcare costs. Finland has a public tax-funded healthcare system, allowing every citizen to be equally treated at the right level of care at every step (including acute care and rehabilitation), independent of for example socioeconomic factors and insurance status. In regard to neurocritical disease, all specialized neurointensive care has been centralized to five university hospitals for several decades. Thus, all the most severe and resource-demanding cases are treated at the university hospital level, enabling comprehensive cost-analysis studies. Furthermore, the Social Insurance Institution (Kela) in Finland covers all Finnish citizens regardless of personal insurance status.

The aim of this study is to describe differences in total one-year healthcare costs and one-year mortality and treatment cost-effectiveness following ICU admission after neurocritical disease (TBI, ICH, SAH and AIS). Especially, we aimed to study treatment cost-effectiveness of independent survivors.

## Methods

### Study design and study population

Finland has a publicly funded three-tier healthcare system, where five university hospitals provide the highest level of care, including specialized neurointensive care. We used the Finnish Intensive Care Consortium (FICC) database to include all adult patients (age ≥ 18 years) treated for TBI, ICH, SAH or AIS in the five university hospitals in Finland from 2003 to 2013. The FICC database is a nationwide prospective data-collecting database including all ICU-treated patients from the majority of all ICUs in Finland [10].

**Fig. 1:**
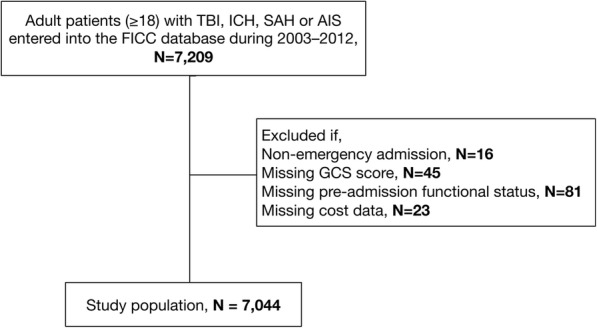
Flow chart. Abbreviations: TBI, traumatic brain injury; ICH, intracerebral hemorrhage; SAH, subarachnoid hemorrhage; AIS, acute ischemic stroke; FICC, Finnish Intensive Care Consortium; GCS, Glasgow Coma Scale

We defined TBI, ICH, SAH and AIS according to the Acute Physiology and Chronic Health Evaluation III (APACHE III) diagnosis and International Classification of Diseases and Related Health Problems, 10th Revision (ICD-10) diagnosis. We confirmed the TBI and ICH diagnoses by reviewing the patients’ computerized tomography (CT) head scans obtained on admission. ICH and SAH diagnoses do not include those due to trauma (these are coded as TBI). We excluded patients with missing baseline data (non-emergency admission, missing Glasgow Coma Scale (GCS) score, missing pre-admission functional status, missing cost data, see Fig. 1).

### Extracted variables

We extracted the following variables from the FICC database: age, GCS score, pre-admission functional status, significant chronic comorbidity, mechanical ventilation, length of ICU stay, length of hospital stay, Therapeutic Intervention Scoring System 76 (TISS-76 [11]), APACHE II [12], Simplified Acute Physiological Score II (SAPS II [13]) and first-day Sequential Organ Failure Assessment (SOFA [14]) score. In FICC, the GCS score is defined as the worst measured GCS score during the first ICU day or the last reliable GCS was used for intubated and/or sedated patients according to the SAPS II definition [13]. Pre-admission functional status is a modified version of the World Health Organization/Eastern Cooperative Oncology (WHO/ECOG) classification used in the FICC [15]. Significant chronic comorbidity is defined according to the APACHE II and SAPS II definitions [12, 13]. The TISS-76 score includes 76 items measuring nursing staff workload and the intensity of given treatments. It was originally developed as a tool to quantify the workload and treatment intensity required by ICU-treated patients, but has since also been used as a dynamic measure of patient prognosis [16].

### Definition of outcome variables

We used one-year mortality and a surrogate marker for permanent disability as outcome variables. We obtained data on date of death through the Finnish population register (available for all patients) and the archive of death certificates. We defined patients permanently disabled if they were granted a permanent disability allowance or disability pension by the Social Insurance Institution (Kela) by 30 September 2016. The criteria for Kela to grant permanent disability allowance or disability pension is being unable to independently carry out daily activities (e.g. self-hygiene, basic housekeeping, take care of things outside the home) or to be unable to return to work, for a minimum of one consecutive year. The Kela criteria for granting permanent disability allowances did not change during the study period [17]. We also report hospital mortality rates.

### Definition of cost variables

The total healthcare cost variable consists of three parameters: university hospital costs, rehabilitation hospital costs and social security costs up to one year after the index admission [17]. We obtained university hospital costs directly from the hospitals’ billing departments. University hospital costs includes expenses from the whole treatment period, including e.g. personnel costs, surgery, ICU stay, laboratory costs, radiological costs, and costs of ward stay. We calculated rehabilitation hospital costs by multiplying the length of rehabilitation stay by the average price of one ward day in the designated level-of-care unit, according to a report from the Finnish National Institute for Health and Welfare [18]. We obtained social security costs directly from Kela. Social security costs include for example disability allowances, sickness allowances, private physician and physiotherapist expenses, prescription drug expenses and medical travel costs. In Finland, Kela is a Finnish government agency funded by tax money, insurance payments, and municipalities that provide all social security payments in Finland. Thus, social security payments are not dependent upon, for example, personal insurance. We adjusted all costs according to the Consumer Price Index (CPI) in Finland to euros based on the currency rate in 2013:

$$ CPI\  adjusted\ cost= Cost\ast \frac{CPI\  in\ 2013}{Admission\ year\  CPI} $$

### Statistics

We used SPSS Statistics 24.0 for mac OS (IBM Corp, Armonk, NY, USA) and Stata Statistical Software for mac OS (StataCorp LP, College Station, TX, USA) for the statistical analyses. We compared categorical variables using the chi-squared (χ^2^) test with the Bonferroni correction. We tested continuous data for skewness and as all variables were highly skewed they are reported as medians with interquartile ranges (IQR), unless other specified (such as cost data). We compared non-parametric continuous variables between groups using a non-parametric test. We present cost data as means with 95% confidence intervals (CI) and compared means between groups using the *t* test.

To compare costs adjusted for severity of illness between the different diagnostic groups we created a severity of illness model using multivariate logistic regression analysis, with one-year mortality and permanent disability (for one-year survivors) as the dependent variable, separately for the diagnostic groups to weight the predictors according to the diagnostic group. The severity of illness model included variables that were significantly (*p* ≤ 0.05) associated with one-year mortality in univariate analysis. These were the same for all diagnoses: age, GCS score, chronic comorbidity, pre-admission functional status and SAPS II score (excluding age, GCS score, and chronic comorbidity). SAPS II was chosen over APACHE II in accordance with findings from our previous studies [19, 20]. The predictive ability of the severity of illness model was assessed by the area under the receiver operating curve (AUC).

We then used multivariate linear regression analysis (*regress* in Stata), adjusting for variables in the severity of illness model to assess differences in costs between the diagnostic groups. We also used the same model in a multilevel mixed-effect logistic regression model (*melogit* in Stata) to assess differences in risk of one-year mortality and permanent disability between the diagnostic groups. Diagnosis (TBI, ICH, SAH, AIS) was modeled as a random effect and considered as the random part of the intercept, as the effect of the included variables may be different in respective diagnostic groups.

To evaluate cost-effectiveness, we calculated the effective cost per survivor (ECPS) and effective cost per independent survivor (ECPIS), which is defined as the cost for all patients divided by the number of one-year survivors or independent one-year survivors [21]. As a sensitivity analysis we divided patients into four risk bands according to probability (0–25%, 26–50%, 51–75%, 76–100%) of one-year mortality within the diagnostic group. We conducted the study according to the Strengthening the reporting of observational studies in epidemiology (STROBE) guidelines (Additional file 1).

## Results

The final study population included 7044 patients (Fig. 1). Of these, 3097 had TBI (43%); 949 had ICH (14%); 1875 had SAH (27%) and 1123 had AIS (16%). Patient baseline characteristics are shown in Table 1. Patients with AIS or ICH were older those with than TBI or SAH, respectively. The GCS score was lower among patients with TBI or ICH compared to SAH or AIS. Patients were predominantly female only in the group of patients with SAH. Patients with SAH had the best pre-admission functional status. There were no major differences in significant comorbidities between the diagnostic groups. Patients with SAH had the longest ICU and hospital length of stay. Mean daily treatment intensity measured using the TISS-76 was similar in patients with TBI, ICH or SAH but lower among patients with AIS. Total treatment intensity was notably higher in patients with SAH compared to the other groups. Patients with ICH had the highest SAPS II and APACHE II scores followed by patients with TBI, SAH or AIS.

**Table 1:** Patient baseline characteristics

Variables	TBI (*N* = 3097)	ICH (*N* = 949)	SAH (*N* = 1875)	AIS (*N* = 1123)	*P* value
Age (median, IQR)	56 (41–67)	61 (52–69)	56 (47–65)	68 (59–76)	< 0.001
18–40 years	750 (24%)	71 (7%)	193 (10%)	42 (4%)	< 0.001
41–64 years	1455 (47%)	509 (54%)	1201 (64%)	420 (37%)	
≥ 65 years	892 (29%)	369 (39%)	481 (26%)	661 (59%)	
GCS score, median (IQR)	9 (5–14)	8 (4–13)	12 (5–15)	12 (8–15)	< 0.001
3–8	1465 (47%)	497 (52%)	704 (38%)	313 (28%)	< 0.001
9–12	598 (20%)	172 (18%)	262 (14%)	253 (22%)	
13–15	1034 (33%)	280 (30%)	909 (48%)	557 (50%)	
Female	711 (23%)	332 (35%)	1065 (57%)	443 (39%)	< 0.001
Pre-admission functional status^a^					< 0.001
Fit for work or equal	1896 (61%)	613 (65%)	1473 (79%)	717 (64%)	
Unfit for work, but independent in self-care	967 (31%)	252 (27%)	332 (18%)	275 (24%)	
Partially dependent in self-care	180 (6%)	61 (6%)	47 (2%)	110 (10%)	
Totally dependent in self-care	54 (2%)	23 (2%)	23 (1%)	21 (2%)	
Significant chronic comorbidity^b^	267 (9%)	108 (11%)	154 (8%)	138 (12%)	< 0.001
Mechanical ventilation	2070 (67%)	640 (67%)	1241 (66%)	427 (38%)	< 0.001
LOS ICU, days (median, IQR)	2 (1–4)	2 (1–3)	3 (1–6)	1 (1–2)	< 0.001
LOS hospital, days (median, IQR)	6 (3–11)	6 (3–12)	10 (6–16)	6 (4–11)	< 0.001
TISS-76 daily average^c^ (median, IQR)	27 (21–33)	26 (20–31)	28 (24–34)	19 (14–27)	< 0.001
TISS-76 total^c^ (median, IQR)	69 (42–154)	63 (39–126)	102 (63–230)	44 (27–74)	< 0.001
APACHE II score (median, IQR)	19 (13–25)	20 (13–26)	17 (11–24)	14 (9–22)	< 0.001
SAPS II score (median, IQR)	35 (24–50)	40 (25–55)	29 (20–48)	29 (23–45)	< 0.001
SOFA score^d^ (median, IQR)	6 (3–8)	6 (3–9)	6 (3–9)	3 (1–7)	< 0.001

*Abbreviations:* AIS, acute ischemic stroke; APACHE, Acute Physiology and Chronic Health Evaluation; GCS; Glasgow Coma Scale; ICH, intracerebral hemorrhage; LOS, length of stay; ICU, Intensive Care Unit; SAH, subarachnoid hemorrhage; SAPS, Simplified Acute Physiology Score; SOFA, Sequential Organ Failure Assessment; TBI, traumatic brain injury; TISS-76, Therapeutic Intervention Scoring System 76

^a^A modified World Health Organization/Eastern Cooperative Oncology Group classification system implemented by the Finnish Intensive Care Consortium

^b^Any chronic comorbidity according to APACHE II or to SAPS II

^c^Missing for 6 patients

^d^Missing for 9 patients

### Severity of illness models

The AUC for the severity of illness model, for one-year mortality prediction, including all patients, was 0.86 (95% CI 0.85–0.87), indicating good discrimination. The AUC was 0.85 (95% CI 0.84–0.87) for patients with TBI, for ICH the AUC was 0.85 (95% CI 0.82–0.87), for SAH the AUC was 0.89 (95% CI 0.87–0.90) and for AIS the AUC was 0.88 (95% CI 0.85–0.90). The AUC for the severity of illness model, for permanent disability prediction (for one-year survivors), including all patients, was 0.71 (95% CI 0.70–0.73), indicating satisfactory discrimination. The AUC was 0.73 (95% CI 0.70–0.75) for patients with TBI, for ICH the AUC was 0.67 (95% CI 0.62–0.71), for SAH the AUC was 0.72 (95% CI 0.69–0.75) and for AIS the AUC was 0.68 (95% CI 0.64–0.72).

**Fig. 2:**
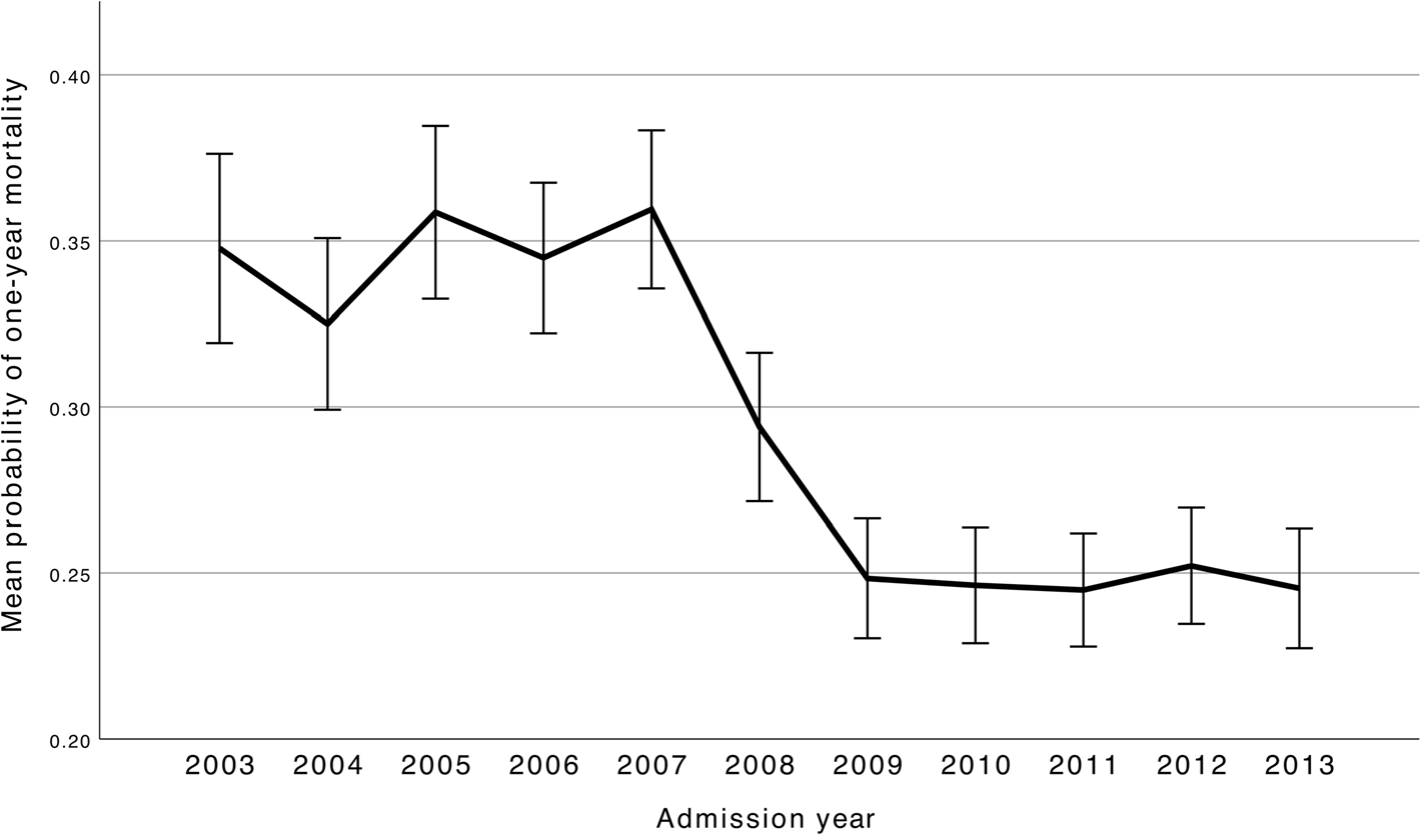
Changes in mean probability of one-year mortality (with 95% confidence intervals), reflecting patient severity of illness. Probabilities are calculated by logistic regression analysis, adjusting for age, Glasgow Coma Scale score, significant comorbidity, pre-admission functional status and the modified Simplified Acute Physiology Score II. The y-axis scale extends from 0 to 0.4, where 0 indicates that the probability is 0% and 0.4 that the probability is 40%. Severity of illness decreased markedly from 2007 to 2009, where after it remained largely the same

The overall probability of one-year mortality, reflecting severity of illness, decreased during the study period (Fig. 2). This decrease was most profound in patients with AIS or ICH (Additional file 2). With time the relative proportion of patients in the two lowest risk bands (0–25%, 26–50% vs. 51–75%, 76–100%) became greater in all diagnostic groups but especially in the ICH and AIS groups (Additional file 3).

### Patient outcome

Patient outcome is shown in Table 2. Hospital mortality was highest among patients with ICH (28%), followed by SAH (18%), AIS (16%) and TBI (13%). One-year mortality was highest among patients with ICH (44%) followed by AIS (29%), SAH (27%) and TBI (25%). In a multilevel mixed-effect logistic regression model, using TBI as the reference group, ICH was associated with the highest risk of one-year mortality (OR 2.61, 95% CI 2.15–3.16) followed by AIS (OR 1.90, 95% CI 1.55–2.33) and SAH (OR 1.79, 95% CI 1.51–2.11).

**Table 2:** Mean costs and unadjusted outcomes by diagnosis

Outcome	TBI (*N* = 3097)	ICH (*N* = 949)	SAH (*N* = 1875)	AIS (*N* = 1123)	*P* value
Hospital mortality	12.5% (11.3–13.6)	27.6% (24.8–30.5)	18.3% (16.5–20.0)	16.4% (14.2–18.6)	< 0.001
One-year mortality	24.7% (23.2–26.2)	43.8% (40.7–47.0)	27.1% (25.1–29.1)	28.7% (26.0–31.3)	< 0.001
Permanent disability^a^	36.7% (34.8–38.7)	51.0% (46.8–55.3)	26.0% (23.7–38.4)	46.9% (43.5–50.4)	< 0.001
Mean costs					
University hospital costs	€19,568 (18,707–20,429)	€18,721 (17,125–20,316)	€25,717 (24,722–26,713)	€15,819 (14,665–16,972)	< 0.001
Percentage of total costs	45%	39%	50%	40%	
Rehabilitation hospital	€18,435 (17,164–19,706)	€21,361 (19,055–23,667)	€16,673 (15,112–18,234)	€16,579 (14,720–18,437)	< 0.001
Percentage of total costs	42%	45%	32%	42%	
Social security costs	€5913 (5608–6217)	€7579 (6791–8367)	€9516 (8975–10,057)	€6824 (6158–7491)	< 0.001
Percentage of total costs	13%	16%	18%	17%	
Total costs	€43,916 (42,096–45,735)	€47,661 (44,198–51,123)	€51,906 (49,544–54,268)	€39,222 (36,541–41,903)	< 0.001

Outcome data presented as percentages with 95% confidence intervals (CI). Cost data presented as means with 95% CIs

*Abbreviations:* AIS, acute ischemic stroke; ICH, intracerebral hemorrhage; SAH, subarachnoid hemorrhage; TBI, traumatic brain injury

Costs shown in euros (€) at the 2013 rate

^a^Permanent disability for one-year survivors

Permanent disability in one-year survivors was highest among patients with ICH (51%), followed by AIS (47%), TBI (37%) and SAH (26%). In the multilevel mixed-effect logistic regression model, using TBI as the reference group, ICH was associated with the highest risk of permanent disability (OR 1.75, 95% CI 1.42–2.14) followed by AIS (OR 1.53, 95% CI 1.27–1.85). In contrast, SAH was associated with a lower risk of permanent disability compared to TBI (OR for SAH in comparison to TBI was 0.75, 95% CI 0.64–0.88).

### Costs and cost-effectiveness

Overall mean cost per patient was €45,799 (95% CI €44,597–€47,001) out of which 45% constituted university hospital costs (mean €20,493, 95% CI 19,945–21,040), 39% rehabilitation hospital costs (mean €18,064, 95% CI €17,245–18,883) and 16% social security costs (mean €7242, 95% CI €6992–€7492).

Differences in costs between the diagnostic groups are shown in Table 2. University hospital costs were notably higher for patients with SAH compared to the other diagnostic groups (€25,717 vs. €15,819–€19,568). Rehabilitation costs were similar between the groups, being slightly higher for patients with TBI or ICH compared to SAH or AIS (€18,435 and €21,361 compared to €16,673 and €16,579). Social security costs were highest for patients with SAH and lowest for patients with TBI (€9516 vs. €5913).

**Fig. 3:**
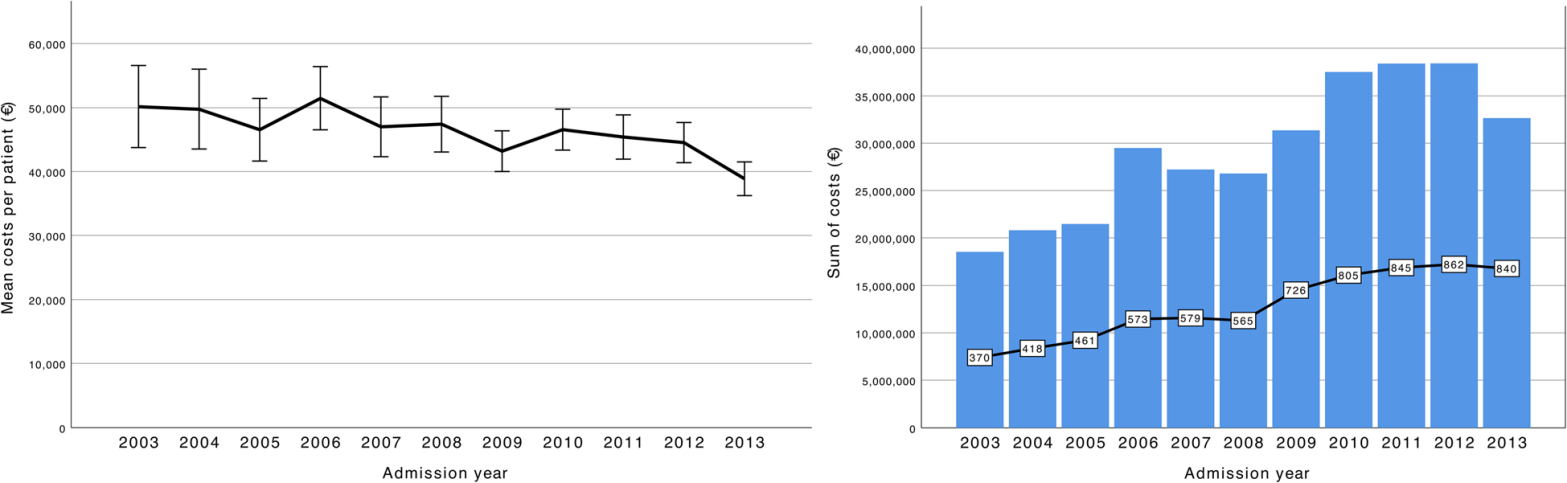
Left panel, changes in mean costs per patient during the study period (with 95% confidence intervals (CI)]). A trend towards lower mean costs per patient is noted. Mean cost per patient was €50,162 (95% CI €43,783–€56,541) in 2003 and €38,872 (95% CI €36,236–€41,508) in 2013. Right panel, changes in the sum of costs (blue bars) and absolute number of patients per year (connected boxes). The sum of costs increased by 76% from 2003 to 2013 (€18.6 million in 2003 and €32.8 million in 2013). The total number of patients increased by 227% from 370 patients in 2003 to 840 patients in 2013

Change in the sum of costs for all patients is shown in Fig. 3. In 2003, the sum of costs for all patients was €18.6 million. In 2013, this sum had risen to €32.7 million or on average by 7% per year. Still, the mean cost per patient decreased on average by 2% from 2003 to 2013 (Fig. 3). Changes in the sum of costs for the different diagnostic groups is shown in Additional file 4. The sum of costs increased notably in all groups with the most marked relative increase in patients with AIS. Additional file 5 shows changes in mean cost per patient during the study period. On average, the mean cost per patient decreased in patients with TBI or ICH, remained unchanged in patients with AIS and increased in patients with SAH.

In the linear regression model, adjusting for the severity of illness model using TBI as the reference population, SAH was associated with the highest costs (€7761, 95% CI €4813–€10,709, *p* < 0.001), followed by ICH (€4044, 95% CI €305–€7782, *p* = 0.034). On the contrary, AIS was associated with a lower total cost compared to TBI (− 4861 euros, 95% CI − 8365 to − 1357 euros, *p* = 0.007).

**Fig. 4:**
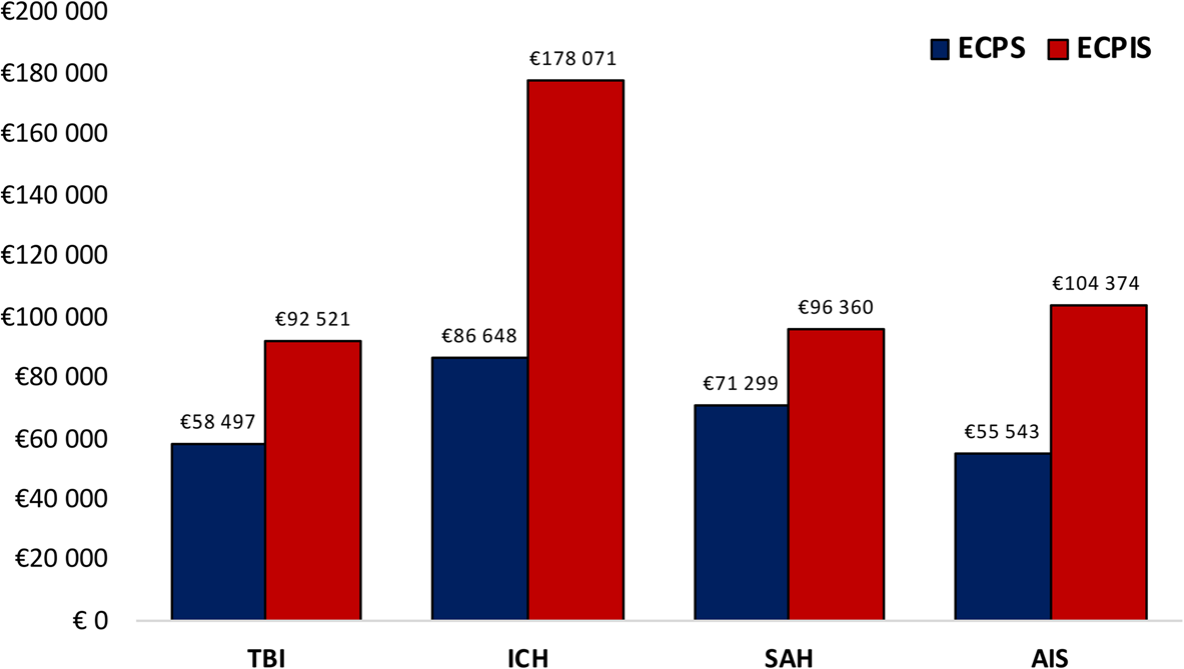
Effective cost per survivor (ECPS) in blue and effective cost per independent survivor (ECPIS) in red

In regard to cost-effectiveness, AIS had the lowest ECPS, followed by TBI, SAH and ICH. However, the ECPIS for TBI and SAH was lower than for AIS and ICH (Fig. 4). In the sensitivity analysis, TBI and SAH had the lowest ECPIS in all risk bands (Additional file 6). In the most severely ill patients in risk band 4, TBI and AIS had the lowest ECPS, while TBI and SAH had the lowest ECPIS. ICH was associated with the lowest cost-effectiveness in all risk bands.

## Discussion

### Principal findings

In this comprehensive cost-analysis study, we found that the burden of ICU-treated TBI, ICH, SAH and AIS has markedly increased during the past decade in Finland. This is mainly due to an increased number of patients requiring neurocritical care being admitted to the ICU, as mean cost per patient slightly decreased during the study period. The decrease in mean cost per patient probably reflects the fact that severity of illness was less in all diagnostic groups, indicating that more patients with less severe neurocritical illness are treated in the ICU nowadays. We also identified major differences in treatment costs and cost-effectiveness in patients with TBI, SAH, ICH or AIS. Intensive care of patients with TBI or SAH resulted more commonly in patients becoming independent survivors and was associated with lower treatment costs compared to ICH and AIS. That said, it should be noted that a direct comparison between patients with TBI, ICH, SAH or AIS is challenging due to the varied spectrum of patient demographics, natural history, ICU admission thresholds, treatment options and strategies and baseline prognosis.

### Comparison with other studies

There are no studies that have comprehensively included several neurocritical illnesses. Regarding ICH and SAH, a recent study from Ontario, showed that total direct and indirect hospital costs for patients with spontaneous ICH was $53,491 (expressed in Canadian dollars at the 2017 rate, converts to €35,303 at the 2013 rate) and $92,794 (expressed in Canadian dollars at the 2017 rate, converts to €61,243 at the 2013 rate) for patients with SAH [22]. The mean cost per SAH hospital survivor was $136,097 (expressed in Canadian dollars at the 2017 rate, converts to €89,797 at the 2013 rate) and the mean cost per ICH hospital survivor was $94,856 (expressed in Canadian dollars at the 2017 rate, converts to €62,585 at the 2013 rate). We found similar results, as the mean cost per one-year ICH was €84,859 (expressed in euros at the 2013 rate) and 71,195 (expressed in euros at the 2013 rate), per one-year SAH survivor.

Regarding SAH, a study from the UK in 2001, showed that the cost per life saved was £40,816 (expressed in pounds sterling at the 2001 rate, converts to €82,546 at the 2013 rate) and the cost to avoid one bad outcome was £84,366 (expressed in pounds sterling at the 2001 rate, converts to €170,561 at the 2013 rate) [23]. Quite similarly, the ECPS in our study was €71,195 (expressed in euros at the 2013 rate) but the ECPIS was markedly lower, being €96,265 (expressed at the 2013 rate). Another study from the UK reported that the estimated cost for treating one patient with SAH was £23,294 (expressed in pounds sterling at the 2005 rate, converts to €38,701 at the 2013 rate). This is somewhat lower than in our study, where the mean cost per patient with SAH was €51,906 (expressed in euros at the 2013 rate). Yet, the UK study did not include, for example, medication costs. In our study, the social security reimbursements were on average €9516 per patient with SAH, making the results between our study and the UK study very similar.

SAH was associated with the highest university hospital costs whereas AIS was associated with the lowest university hospital costs. In contrast to the other diagnostic group, most patients with SAH undergo extensive and costly treatments, including aneurysm clipping or endovascular treatment [24]. The low university hospital costs in the AIS group may plausibly be explained by less severe illness (measured by the APACHE II and SAPS II), fewer patients being mechanically ventilated, shorter lengths of stay and lower treatment intensity (measured by the TISS-76). SAH, on the other hand, was associated with the highest treatment intensity and longest length of stay, leading to higher university hospital costs. This and our previous study provide good data on the cost-effectiveness of intensive care in TBI [17]. Other TBI studies have identified total one-year direct and indirect costs between €16,579 and €35,560 (converted to euros at the 2013 rate) [25–27] but data on cost-effectiveness are lacking.

An inbuilt study bias is that ICU admission criteria vary among the diagnostic groups included. For example, patients with SAH are preferably directly admitted to the ICU in some centers, independent of their level of consciousness, while patients with TBI, ICH or AIS can be treated outside of the ICU, given an adequate level of consciousness and appropriate radiological findings. Further, patient age is probably key in ICU selection criteria, as elderly patients with stroke are often excluded from aggressive ICU treatment. This may distort our results towards improved cost-effectiveness, as some patients are admitted due to an estimated favorable outcome. It should also be mentioned that some of the patients included might be admitted to ICU due to potential organ donation. The American Heart Association/American Stroke Association recommends full treatment for at least 2 days before implementing any treatment limitations [28]. Organ donors often receive aggressive ICU treatment to uphold adequate organ vitality, which thus, is very resource-demanding as well. However, assessing the potential ICU treatment cost-effectiveness benefits of organ donation is out of the scope of this study.

We noted a marked decrease in unadjusted one-year mortality between 2007 and 2009. During the same years a substantial increase in patients admitted with ICH or AIS were noted due to structural changes in some centers leading to more less severely ill patients being admitted, which most likely explains the noted shift in mortality. It should also be highlighted that the vast majority of patients with ICH or AIS are treated outside of the ICU setting (for example in stroke units). In our study setting, only patients with the most severe cases of ICH and AIS are treated in the ICU, as all hospitals included have dedicated stroke units. Thus, our study obviously undervalues the total burden of AIS and ICH but represent the ICU-treated portion well. Furthermore, during the study period thrombectomy had not been established in the treatment of large vessel occlusion AIS [29–33]. The patients with and large vessel occlusion AIS probably constitute a notable proportion of patients with AIS being treated in the ICU prior to the establishment of thrombectomy. Thus, considering the effectiveness of thrombectomy for large vessel occlusion, the long-term cost-effectiveness of AIS has most likely improved and the number of patients with AIS requiring ICU care at all have may have decreased [34, 35]. This limits the interpretation and the generalization of the AIS results to the modern thrombectomy era. Cost-effectiveness correlated strongly with the severity of illness and the ECPIS was highest among patients with ICH or AIS in all risk bands, with the exception of risk band 4, where AIS was associated with the lowest ECPIS. Although we cannot confirm this, it is possible that these patients represent a group with relatively small infarcts but severe extracranial organ dysfunction (e.g. respiratory dysfunction after basilar artery occlusion), requiring intensive care, from which recovery is favorable.

### Future directions

In our study we noted a steady increase in the number of neurocritical patients treated in the ICU. This probably reflects the parallel increase in ICU beds and the rapidly aging population in Finland, increasing the need for intensive care [8]. The total number of ICU beds in Finland is approximately 6 per 100,000 persons, which is among the lowest in Europe and one fifth of that in the USA [36, 37]. Thus, considering the rapidly aging population a continued increase in the number of patients requiring neurocritical care and an increased demand for ICU beds are to be expected. Accordingly, efforts should be put towards preventing these events, as outcomes are poor and treatment is resource-demanding and costly. Effectively treating risk factors for stroke (SAH, ICH, AIS) and TBI has the potential to reduce the burden of these neurocritical illnesses. For example, the decreased incidence of smoking in Finland has been linked to a decreased incidence in SAH [38]. Similar prevention should be targeted in TBI, which could be achieved by avoiding falls among the elderly, and reducing alcohol-related falls and traffic-related accidents.

### Strengths and limitations

This study was conducted in a government tax-funded healthcare system, where all citizens have the right to equal care, avoiding selection bias due to differences in access to treatment. Further, specialized neurointensive care has for decades been centralized to five university hospitals and, thus, the current study represents as good as the whole Finnish adult population. Moreover, data on rehabilitation hospital length of stay is electronically recorded on a national level by the Finnish National Institute for Health and Welfare, making this data extremely comprehensive. Another unique feature of this study is that in Finland, social security costs are essentially paid by one institution, Kela, which covers all Finnish citizens regardless of insurance status. Thus, in combination with the high-quality FICC database with the small number of missing patients, our study comprehensively catches the burden of neurocritical illnesses treated in the ICU in a developed country setting [10].

Regarding patients with TBI or SAH, we were able to include four of five ICUs providing specialized intensive care for these patients, making the TBI and SAH largely representative of the whole Finnish population, although less so than for ICH and AIS. Yet, as already mentioned, not all patients with AIS or ICH are treated in the ICU. After the introduction of thrombectomy the number of patients with AIS requiring ICU care has probably decreased as well. The noted increase in AIS admissions in this study is due to a change in local policy in one of the hospitals leading to more patients with AIS with less severe illness being treated in the ICU, probably explaining the decrease in unadjusted mortality in the middle of the study period. Thus, clearly our results on patients with AIS or ICH cannot be generalized to patients being treated outside the ICU.

We used a surrogate marker of permanent disability, i.e. if the patient was granted a permanent disability allowance or disability pension by Kela. In comparison to other commonly used outcome scales (e.g. Glasgow Outcome Scale, modified Rankin Scale), our definition may include some degree in inaccuracy but considering that all Finnish citizens are covered by Kela we believe that our definition adequately reflects neurological outcome. Further, the FICC database does not contain uniformly standardized data on limitation of care from the beginning of the study period. Thus, some included patients are probably ICU-treated due to upcoming potential organ donation.

We did only study adult patients and, thus, our results cannot directly be generalized to the pediatric population. Further, we only included patients with TBI, ICH, SAH or AIS. Thus, other neurocritical illnesses, such as cerebral venous thrombosis, status epilepticus, bacterial meningitis, viral encephalitis and neuromuscular disorders were not included. Last, the study was conducted in a single Nordic country. Thus, our results are best applicable to settings with similar healthcare systems.

## Conclusion

Neurocritical illnesses are costly and resource-demanding diseases associated with poor outcomes. The total number of patients requiring neurocritical care who are admitted to the ICU has increased leading to an increase in total costs, although mean cost per patient has remained largely unchanged. These changes were paralleled with a decrease in patients’ severity of illness. Intensive care of patients with TBI or SAH more commonly resulted in them becoming independent survivors and was associated with lower costs compared to the care of patients with ICH or AIS.

## Additional files

Additional file 1: The STROBE guidelines. (DOC 84 kb)

Additional file 2: Changes in probability of one-year mortality (with 95% confidence intervals), reflecting severity of illness, for the diagnostic groups. The y-axis scale extends from 0 to 0.8, where 0 indicates that the probability is 0% and 0.8 that the probability is 80%. Probabilities were calculated by logistic regression analysis adjusting for age, GCS score, chronic comorbidity, pre-admission functional status and SAPS 2, separately for the diagnostic groups. A trend towards lower severity of illness was noted in all diagnostic groups. A small reduction in severity of illness was noted for patients with traumatic brain injury and subarachnoid hemorrhage. Between 2007 and 2009, severity of illness dropped notably in patients with acute ischemic stroke and intracerebral hemorrhage. (TIF 323 kb)

Additional file 3: Temporal change in risk bands within the diagnostic groups. Patients were divided into 4 equally sized risk bands within their own diagnostic group according to risk of one-year mortality (severity of illness). The relative proportion of patients in risk bands 1 and 2 increased with time in all diagnostic groups. The increase was most notable among patients with ICH and AIS, indicating that more patients with less severe illness were admitted towards the end of the study period. (TIF 544 kb)

Additional file 4: Changes in the sum of costs and absolute number of patients during the study period by diagnostic group. The bars (blue) represent the sum of costs for all patients in the particular diagnostic group treated during that particular year. The connected boxes indicate the absolute number of patients in the particular diagnostic group treated during that particular year. The absolute number of patients treated in the intensive care unit increased in all diagnostic groups. Accordingly, the sum of costs increased. (TIF 3003 kb)

Additional file 5: Changes in mean cost per patient (with 95% confidence intervals) during the study period for the diagnostic groups. A trend towards lower mean cost per patient was noted in the traumatic brain injury, intracerebral hemorrhage and acute ischemic stroke groups, while mean cost per patient remained largely the same for patients in the subarachnoid hemorrhage group. (TIF 1051 kb)

Additional file 6: Effective cost per survivor (ECPS) and effective cost per independent survivor (ECPIS) according to diagnostic group and risk band. Risk band 1 (upper left) represents patients with one-year mortality risk of 0–25%, risk band 2 (upper right) represents patients with one-year mortality risk of 26–50%, risk band 3 (lower left) represents patients with one-year mortality risk of 51–75%, and risk band 4 represent patients with one-year mortality risk of 76–100%. The risk bands were created separately for all diagnostic groups by adjusting for age, GCS score, pre-admission functional status, significant chronic comorbidity and the modified SAPS II score. (TIF 931 kb)

## Abbreviations

AIS: Acute ischemic stroke
APACHE II: Acute Physiology and Chronic Health Evaluation II
AUC: Area under the receiver operating characteristic curve
CPI: Consumer Price Index
CT: Computed tomography
ECOG: Eastern Cooperative Oncology
ECPIS: Effective cost per independent survivor
ECPS: Effective cost per survivor
FICC: Finnish Intensive Care Consortium
GCS: Glasgow Coma Scale
ICH: Intracerebral hemorrhage
SAH: Subarachnoid hemorrhage
SAPS II: Simplified Acute Physiology Score II
SOFA: Sequential Organ Failure Assessment
STROBE: Strengthening the reporting of observational studies in epidemiology
TBI: Traumatic brain injury
TISS-76: Therapeutic Intervention Scoring System-76
WHO: World Health Organization
