# Supplementary figures and images for: Costs, outcome and cost-effectiveness of neurocritical care: a multi-center observational study
Source: Crit Care. 2018 Sep 20;22:225. doi: 10.1186/s13054-018-2151-5 (PMC6148794; doi:10.1186/s13054-018-2151-5)

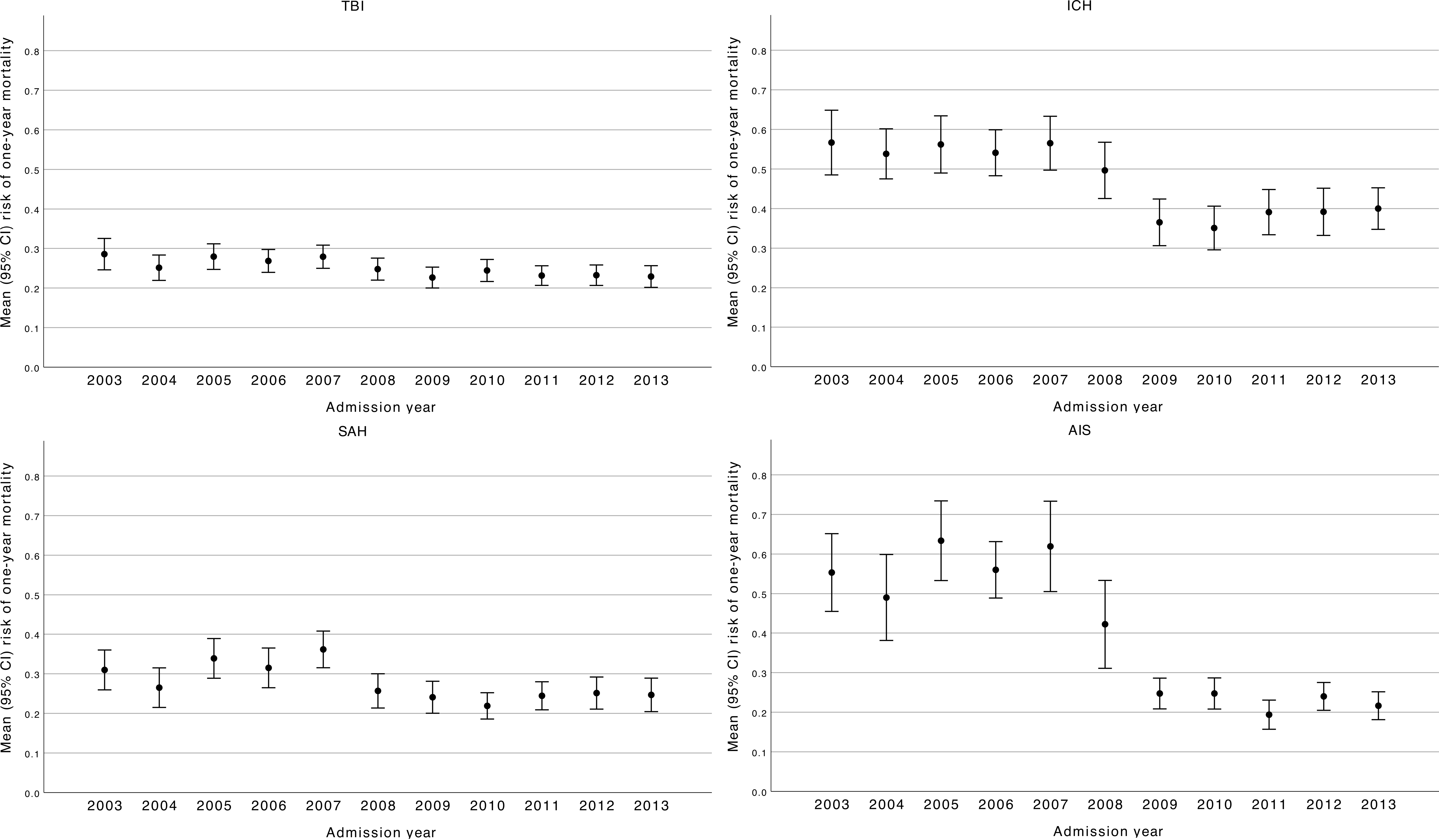

Supplement: Supplementary file 2 — Changes in probability of one-year mortality (with 95% confidence intervals), reflecting severity of illness, for the diagnostic groups. The y-axis scale extends from 0 to 0.8, where 0 indicates that the probability is 0% and 0.8 that the probability is 80%. Probabilities were calculated by logistic regression analysis adjusting for age, GCS score, chronic comorbidity, pre-admission functional status and SAPS 2, separately for the diagnostic groups. A trend towards lower severity of illness was noted in all diagnostic groups. A small reduction in severity of illness was noted for patients with traumatic brain injury and subarachnoid hemorrhage. Between 2007 and 2009, severity of illness dropped notably in patients with acute ischemic stroke and intracerebral hemorrhage. (TIF 323 kb) [file 13054_2018_2151_MOESM2_ESM.tif]

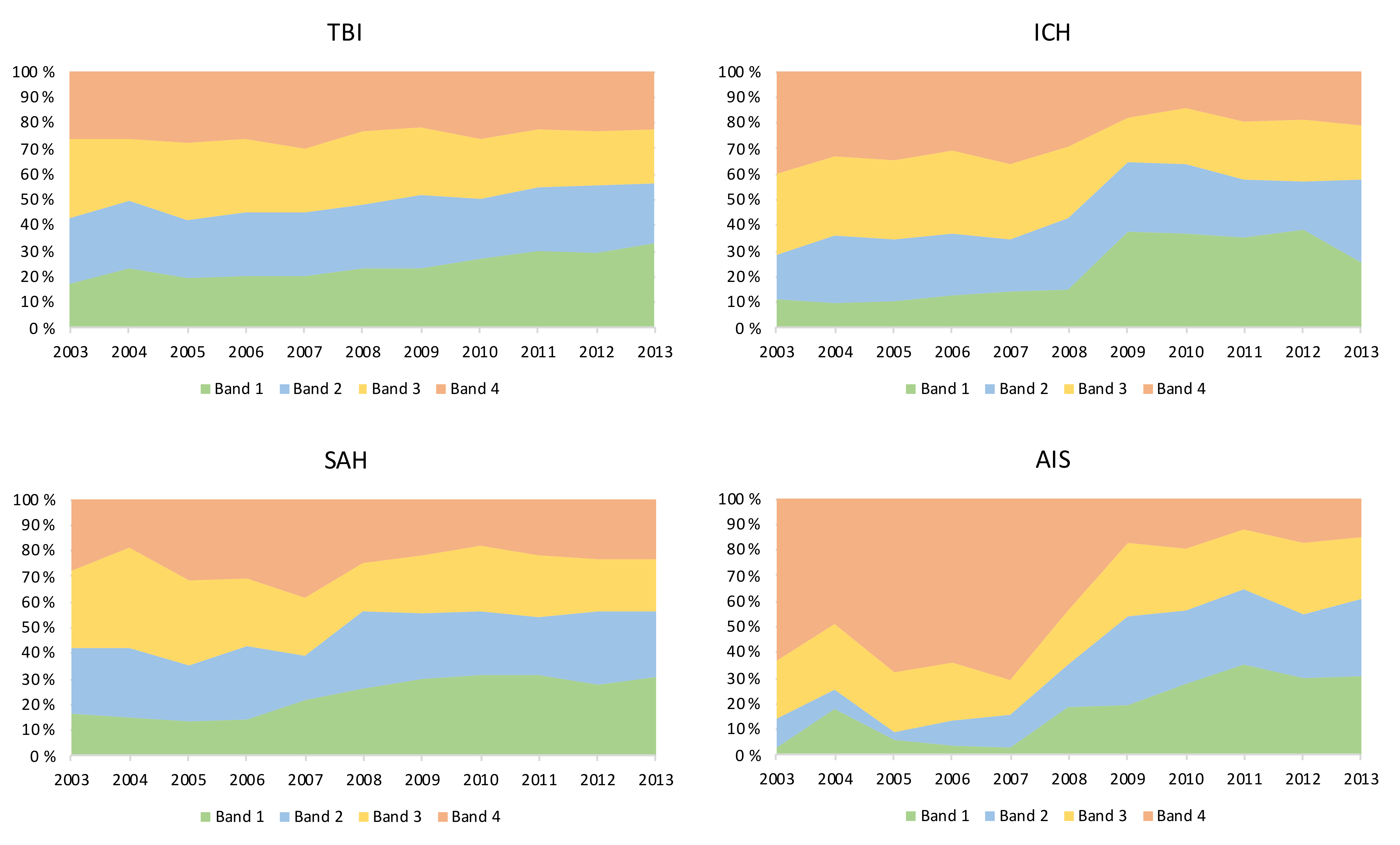

Supplement: Supplementary file 3 — Temporal change in risk bands within the diagnostic groups. Patients were divided into 4 equally sized risk bands within their own diagnostic group according to risk of one-year mortality (severity of illness). The relative proportion of patients in risk bands 1 and 2 increased with time in all diagnostic groups. The increase was most notable among patients with ICH and AIS, indicating that more patients with less severe illness were admitted towards the end of the study period. (TIF 544 kb) [file 13054_2018_2151_MOESM3_ESM.tif]

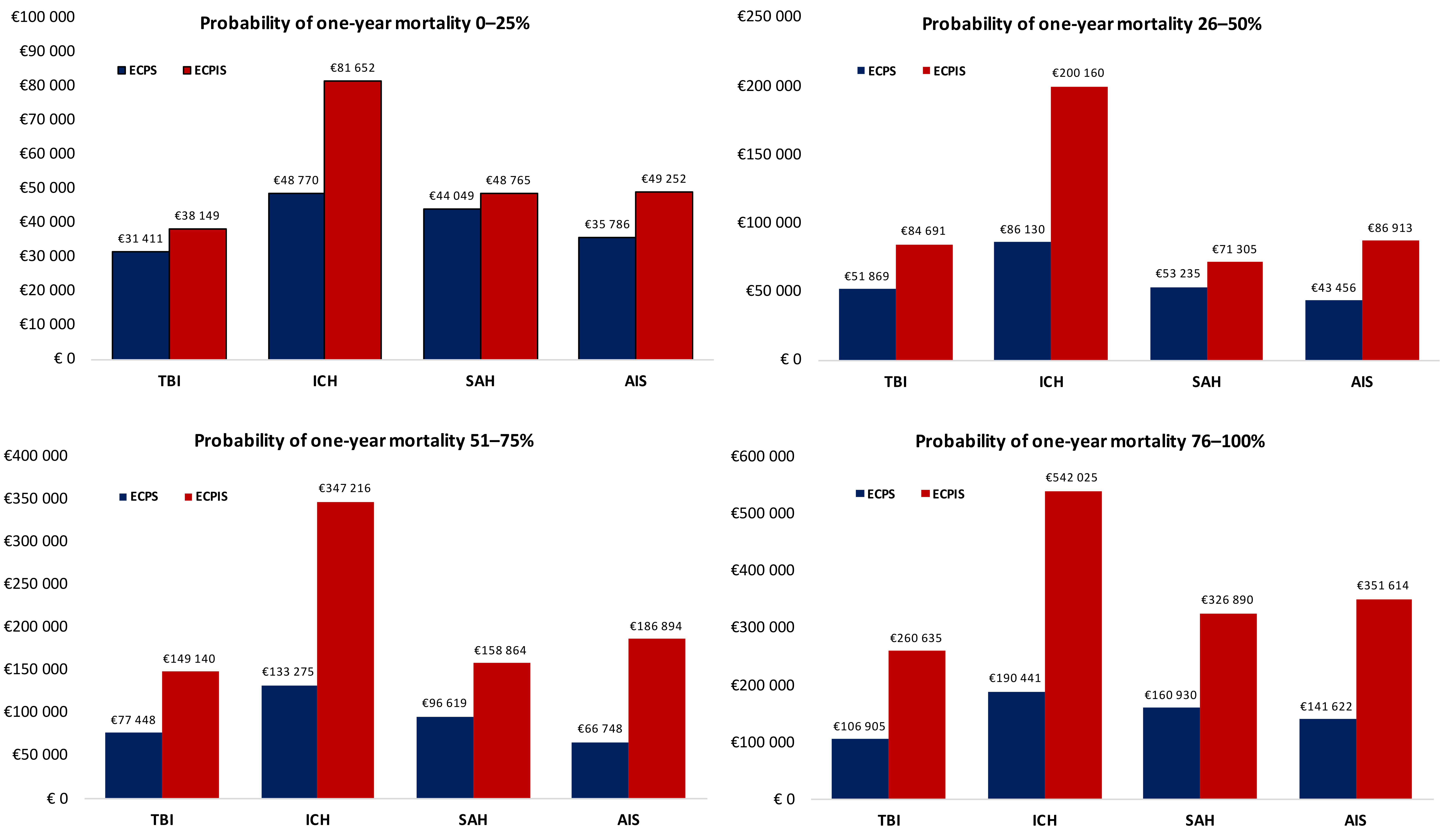

Supplement: Supplementary file 6 — Effective cost per survivor (ECPS) and effective cost per independent survivor (ECPIS) according to diagnostic group and risk band. Risk band 1 (upper left) represents patients with one-year mortality risk of 0–25%, risk band 2 (upper right) represents patients with one-year mortality risk of 26–50%, risk band 3 (lower left) represents patients with one-year mortality risk of 51–75%, and risk band 4 represent patients with one-year mortality risk of 76–100%. The risk bands were created separately for all diagnostic groups by adjusting for age, GCS score, pre-admission functional status, significant chronic comorbidity and the modified SAPS II score. (TIF 931 kb) [file 13054_2018_2151_MOESM6_ESM.tif]
